# Columnar Segregation of Magnocellular and Parvocellular Streams in Human Extrastriate Cortex

**DOI:** 10.1523/JNEUROSCI.0690-17.2017

**Published:** 2017-08-16

**Authors:** Roger B.H. Tootell, Shahin Nasr

**Affiliations:** ^1^Martinos Center for Biomedical Imaging, Charlestown, Massachusetts 02129, and; ^2^Department of Radiology, Harvard Medical School, Boston, Massachusetts 02115

**Keywords:** color, contrast sensitivity, cortical hierarchy, high-resolution fMRI, spatial frequency, stereopsis

## Abstract

Magnocellular versus parvocellular (M-P) streams are fundamental to the organization of macaque visual cortex. Segregated, paired M-P streams extend from retina through LGN into V1. The M stream extends further into area V5/MT, and parts of V2. However, elsewhere in visual cortex, it remains unclear whether M-P-derived information (1) becomes intermixed or (2) remains segregated in M-P-dominated columns and neurons. Here we tested whether M-P streams exist in extrastriate cortical columns, in 8 human subjects (4 female). We acquired high-resolution fMRI at high field (7T), testing for M- and P-influenced columns within each of four cortical areas (V2, V3, V3A, and V4), based on known functional distinctions in M-P streams in macaque: (1) color versus luminance, (2) binocular disparity, (3) luminance contrast sensitivity, (4) peak spatial frequency, and (5) color/spatial interactions. Additional measurements of resting state activity (eyes closed) tested for segregated functional connections between these columns. We found M- and P-like functions and connections within and between segregated cortical columns in V2, V3, and (in most experiments) area V4. Area V3A was dominated by the M stream, without significant influence from the P stream. These results suggest that M-P streams exist, and extend through, specific columns in early/middle stages of human extrastriate cortex.

**SIGNIFICANCE STATEMENT** The magnocellular and parvocellular (M-P) streams are fundamental components of primate visual cortical organization. These streams segregate both anatomical and functional properties in parallel, from retina through primary visual cortex. However, in most higher-order cortical sites, it is unknown whether such M-P streams exist and/or what form those streams would take. Moreover, it is unknown whether M-P streams exist in human cortex. Here, fMRI evidence measured at high field (7T) and high resolution revealed segregated M-P streams in four areas of human extrastriate cortex. These results suggest that M-P information is processed in segregated parallel channels throughout much of human visual cortex; the M-P streams are more than a convenient sorting property in earlier stages of the visual system.

## Introduction

**Figure 1.**
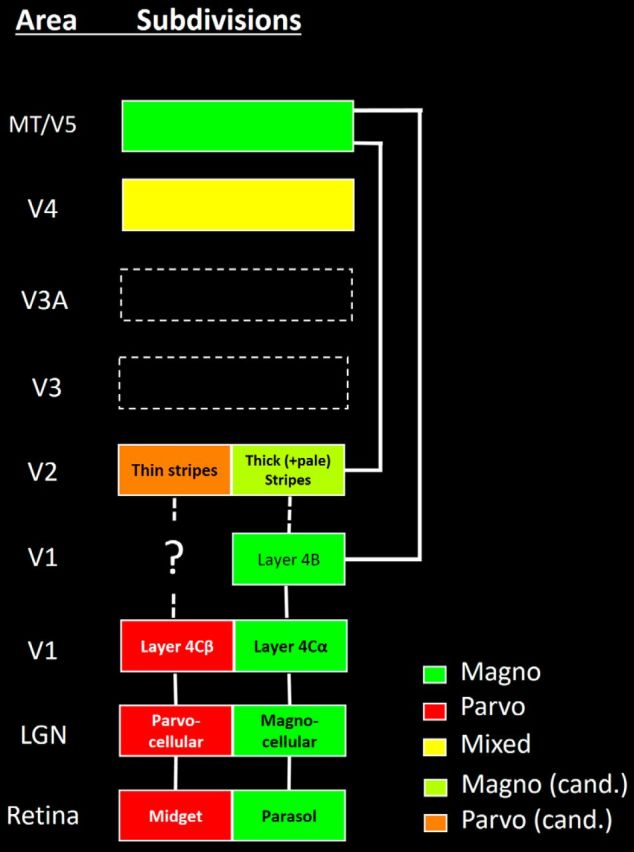
Diagram of M-P streams, based on prior data from macaque monkeys. Color coding and confidence about M-P biases are indicated at the bottom right. cand., Candidate. Diagram represents only a subset of the known connections between areas, which are relevant to ascending M-P streams.

In macaque monkeys, different visual features are processed in functionally and anatomically segregated streams. A prominent example includes the magnocellular versus parvocellular (M-P) streams, which extend in parallel from retina through early visual cortex (Fig. 1) (Hubel and Livingstone, 1987; Zeki and Shipp, 1988; Felleman and Van Essen, 1991; Merigan and Maunsell, 1993). Briefly, M and P ganglion cells (morphologically parasol and midget, respectively) drive neurons within the eponymous magnocellular and parvocellular layers of the LGN. The M and P LGN layers project into V1 layers 4Cα, and 4Cβ, respectively. From V1 to V2, it is controversial whether M and P streams remain segregated and parallel (Hubel and Livingstone, 1987; Livingstone and Hubel, 1987) or whether they are rerouted into a different set of segregated projections, the blobs/patches and interblobs/interpatches (Sincich and Horton, 2002, 2005a, b).

Elsewhere in visual cortex, it also remains unresolved whether M- and P-derived information remains segregated (DeYoe and Van Essen, 1985; Shipp and Zeki, 1985) or whether/where it becomes intermixed (Ferrera et al., 1994). At issue is whether the M and P information in early stages represents (1) two fundamentally different types of visual information (implied by a hypothetical middle-cortical segregation) or (2) an initial organizational stage before subsequent processing along quite different processing dimensions (implied by a cortical intermixing of M and P streams).

Additional questions arise because our current understanding of the M-P pathways is based on data from nonhuman primates. Comparable evidence from humans has been largely nonexistent, partly due to prior technical limitations. Hypothetical M-P streams in humans are likely segregated in thin layers and/or small cortical columns, which are difficult to spatially resolve with conventional fMRI.

However, recent high-resolution fMRI techniques may distinguish M and P layers in human LGN (Denison et al., 2014). Here we used high-resolution fMRI techniques, and a high field (7T) scanner, to test for a column-based segregation of human M-P streams in visual cortical areas V2, V3, V3A, and V4.

Our experiments included both stimulus-dependent and stimulus-independent fMRI measurements. Stimulus-dependent experiments tested for selective cortical responses to signature stimuli that are known to differentiate responses in M-P streams in macaque, including the following: (1) sensitivity to color versus luminance (higher to color in P layers of LGN) (De Valois et al., 1966; Padmos and Norren, 1975; Hicks et al., 1983); (2) sensitivity to binocular disparity, which is prominent in M-dominated cortical area MT (Maunsell and van Essen, 1983; DeAngelis et al., 1998), and thick stripes in V2 (Hubel and Livingstone, 1987; Ts'o et al., 2001; Chen et al., 2008); (3) higher contrast sensitivity and more saturated contrast gain in M (compared with P) stream in retina (Kaplan and Shapley, 1986), LGN (Kaplan and Shapley, 1982; Schiller and Colby, 1983; Derrington and Lennie, 1984; Hawken and Parker, 1984) and V1 (Blasdel and Fitzpatrick, 1984; Tootell et al., 1988a); (4) higher peak spatial frequency tuning (related to smaller RF size) in P (vs M) layers in LGN (Hicks et al., 1983; Derrington and Lennie, 1984) and V1 (Blasdel and Fitzpatrick, 1984); and (5) an interaction of color/luminance sensitivity with spatial frequency sensitivity in P (but not M) layers in LGN; specifically, spatial frequency sensitivity can be lower to color-varying stimuli compared with luminance-varying stimuli, reflecting the receptive field organization of different cone inputs (Hicks et al., 1983; Reid and Shapley, 2002; Solomon and Lennie, 2007). When a given set of extrastriate columns responded selectively to either of these signature stimuli, we interpreted that as additional evidence for a segregation of M-P information in human cortex. These columns included color- and disparity-selective (thin- and thick-type) stripes described previously in V2 and V3 (Nasr and Tootell, 2016; Nasr et al., 2016). Here we further show similarly segregated columns in V4.

A sixth, stimulus-independent experiment tested for intrinsically selective functional connections between each of these column types, during fMRI with eyes closed.

## Materials and Methods

### 

#### 

##### Participants.

A total of eight human subjects (4 females), 22–35 years of age, participated in Experiments 1, 2, and 6. Six of these subjects participated in Experiments 3–5. All subjects had normal or corrected-to-normal visual acuity, color vision (Ishihara test), and radiologically normal brains, without history of neuropsychological disorder. All experimental procedures conformed to National Institutes of Health guidelines and were approved by Massachusetts General Hospital protocols. Written informed consent was obtained from all subjects before the experiments.

All data from areas V3A and V4 are newly reported here, in all subjects, in all experiments. Also in all subjects, all data from our main experiments on M-P stimulus sensitivity (Experiments 3–5) is newly reported. Six of the 8 subjects tested here had participated in a prior experiment on the topography of stripe activity, limited to areas V2 and V3 (Nasr et al., 2016). Data from those 6 original subjects contributed to current results (based on 8 subjects) in areas V2 and V3 (only), in Experiments 1 and 2 (analyses for localization, consistency across sessions and spatial shuffling), and Experiment 6 (functional connections).

##### General procedures.

Each subject was scanned in a 7T scanner, in multiple sessions, on different days. Experiment 1 localized sites selective for (1) color (vs luminance) variations, including thin-type stripes/columns. Experiment 2 localized sites selective for binocular disparity, including thick-type stripes/columns (Nasr et al., 2016). Experiments 3–5 tested additional stimulus parameters, including spatial selectivity and contrast. Experiment 6 mapped functional connectivity of these disparity- and color-selective stripes/columns. In an additional session, all subjects were also scanned in a 3T scanner for structural and retinotopic mapping.

##### Visual stimuli.

Stimuli were presented via a LCD projector (1024 × 768 pixel resolution, 60 Hz refresh rate) focused on a rear-projection screen, viewed through a mirror mounted on the receive coil array. MATLAB, 2013a (The MathWorks) and Psychophysics Toolbox (Brainard, 1997; Pelli, 1997) were used to control stimulus presentation.

During all experiments, stimuli were presented in a blocked design procedure. During stimulus-driven Experiments 1–5, subjects were required to fixate a small (0.1° × 0.1°) central fixation spot. To control the level of attention during the functional scans, subjects conducted an unrelated dummy attention task. Using a response keypad in the scanner, subjects discriminated either (1) a color change between red to green, or vice versa (Experiments 1 and 2), or (2) a shape change between square to circle, or vice versa (Experiments 3–5).

##### Retinotopic visual area localizers.

To localize the retinotopically organized areas V2, V3, V3A, and V4, we presented retinotopic stimuli, as described previously (Nasr et al., 2011). Briefly, stimuli were colored images of real-life scenes, objects, and faces, which were presented within retinotopically limited apertures, against a gray background. In different blocks, the retinotopic apertures were confined to wedges that were centered along the horizontal and vertical meridians (radius = 10°, polar angle = 30°), a foveal disk (radius = 0°-1.5°), or a peripheral ring (radius = 5°–10°). As reference data in some subjects (*n* = 3), the border locations were confirmed based on phase-encoded retinotopic maps (Sereno et al., 1995; Engel et al., 1997; Tootell et al., 1997).

##### Experiments 1 and 2: color selectivity and disparity selectivity.

Data from Experiments 1 and 2 localized color- and disparity-selective stripes and columns, respectively. Further details are furnished below and reported previously (Nasr et al., 2016).

##### Experiment 1: color versus luminance.

Here and throughout Experiments 1–5, experimental stimuli extended 26.7 × 20° in the visual field. To localize V2 color-selective thin-type stripes/columns, Experiment 1 presented sinusoidal gratings that varied in either color or achromatic luminance, in independent blocks (Nasr et al., 2016). In different blocks, grating stimuli were presented at different orientations (0°, 45°, 90°, or 135°), drifting in orthogonal directions (reversed every 6 s) at 4°/s. In each run, these blocks included 9 stimulus presentation blocks (24 s per block). Each run began and finished with an additional block (12 s) of uniform gray of equal mean luminance. Each subject participated in 1 or 2 scan sessions, including 12 runs (1008 functional volumes) per session.

##### Experiment 2: horizontal binocular disparity (3D) versus 2D control.

Thick-type stripes/columns were localized using random dot stereograms (RDSs) (Julesz, 1971; Tsao et al., 2003; Minini et al., 2010; Anzai et al., 2011; Nasr et al., 2016) based on red or green dots (0.09° × 0.09°) presented against a black background. Subjects viewed the stimulus through custom anaglyph spectacles using a Kodak Wratten filter No. 25 (red) over one eye and 44A (cyan) over the other. All subjects reported that stimuli formed a stereoscopic percept of a regular array of cuboids that varied sinusoidally in depth (i.e., ±0.22°), with independent phase. Activity evoked by these stimuli was compared relative to the activity evoked by RDSs (as in the stereoscopic stimuli, but here identical in each eye), in which the fused percept was limited to the frontoparallel plane (i.e., zero depth). Each run included 8 blocks, plus two additional shorter (12 s) blocks of uniform black, at the beginning and end of each run. Each subject participated in 2 or 3 scan sessions (12 runs per session); 864 functional volumes were collected in each of these scan sessions.

##### Experiments 3 and 4: luminance contrast and spatial frequency.

These two experiments were based on a common dataset, which independently varied spatial frequency and contrast. Subjects were presented with gratings of differing achromatic contrast (1.43%, 5.25%, 15.95%, 50.14%, and 99. 62%) and spatial frequency (0.1, 0.27, 0.73, 2.08, and 5.79 cycles/degree) across different blocks, in a 5 × 5 design. Every 4 s, grating orientation changed pseudo-randomly. In addition, grating phase reversed every 1 s, and grating phase was systematically varied. Each run included 15 blocks of 16 s each, beginning and ending with an additional block (12 s) of uniform gray, of equivalent mean luminance. Each subject participated in 2 scan sessions, with 12 runs (1056 functional volumes) per session. Experiment 3 analyzed variations in contrast, combining spatial frequency. Experiment 4 did the reverse.

##### Experiment 5: checkerboard stimuli.

Subjects were presented with color-varying and achromatic luminance-varying checkerboards. For both of these stimulus types, the display was divided into the following number of checks, in *x* and *y* axes, respectively: 1 × 1, 4 × 3, 8 × 6, 16 × 12, and 32 × 24 (i.e., square checks of 20, 6.67, 3.33, 1.67, and 0.83°/side) across different blocks in a 2 × 5 design. Before scanning, the luminance of color-varying stimuli was equated based on flicker photometry (30 Hz), measured (and corrected) across different eccentricities (Nasr et al., 2016). The stimuli were contrast-reversed (phase-reversed) at 1 Hz.

Each run contained 10 blocks (24 s per block), beginning and ending with additional blocks (each = 12 s duration) in response to a baseline stimulus of spatially uniform gray, of luminance equal to that of the checkerboard stimuli. Each subject participated in 1 scan session, including 12 runs (1056 functional volumes) per session.

##### Experiment 6: functional connectivity.

For each subject, data analyzed for functional connectivity were acquired during 4 additional runs of 9 min each, in the 7T scanner; 540 functional volumes were collected from each subject for this experiment. Subjects were asked to keep their eyes closed throughout the functional scans, and to keep their head still. No other instruction was provided.

##### Imaging, 7T.

Except for the retinotopic localizers (see below), all fMRI data were acquired in a 7T Siemens whole-body scanner equipped with SC72 body gradients (70 mT/m maximum gradient strength and 200 T/m/s maximum slew rate) using a custom-built 32-channel helmet receive coil array and a birdcage volume transmit coil (Keil et al., 2010). Single-shot gradient-echo EPI was used to acquire functional images with the following protocol parameter values: TR = 3000 ms, TE = 28 ms, flip angle = 78°, matrix = 192 × 192, BW = 1184 Hz/pix, echo-spacing = 1 ms, 7/8 phase partial Fourier, FOV = 192 × 192 mm, 44 oblique-coronal slices, acceleration factor *r* = 4 with GRAPPA reconstruction and FLEET-ACS data (Polimeni et al., 2016) with 10° flip angle. Voxel size was nominally 1.0 mm, isotropic. The FOV included occipital cortical areas V1, V2, V3, V3A, and V4, but it did not include MT/V5.

During the resting-state scans (Experiment 6), fMRI brain activity fluctuations were measured using a similar EPI protocol, but with TR = 4000 ms, flip angle = 85°, and 59 oblique-coronal slices. Here again, the FOV included areas V1, V2, V3, V3A, and V4.

##### Imaging, 3T.

For practical reasons, retinotopic mapping was conducted using a 3T Siemens scanner (Tim Trio) using a Siemens 32-channel receive coil array. That functional data were acquired using single-shot gradient-echo EPI, with nominally 3.0 mm isotropic voxels, using the following protocol parameters: TR = 2000 ms, TE = 30 ms, flip angle = 90°, matrix = 64 × 64, BW = 2298 Hz/pix, echo-spacing = 0.5 ms, no partial Fourier, FOV = 192 × 192 mm, 33 axial slices covering the entire brain, and no acceleration. As expected, comparison of these retinotopic maps (3T) to the maps collected in a 7T scanner showed a relatively higher spatial resolution in the 7T retinotopic maps, but otherwise the same borders at both field strengths, because most retinotopic borders are fairly smooth across the cortical surface, and large in scale (i.e., long in the representation of polar angle, the retinotopic dimension that distinguishes these area borders).

Structural (anatomical) data were also acquired in a 3T scanner using a 3D T1-weighted MPRAGE sequence with the following protocol parameter values: TR = 2530 ms, TE = 3.39 ms, TI = 1100 ms, flip angle = 7°, BW = 200 Hz/pix, echo spacing = 8.2 ms, voxel size = 1.0 × 1.0 × 1.33 mm, FOV = 256 × 256 × 170 mm.

##### Experimental design and statistical analysis.

The design of this study included both within- and grouped-subject components. Details of the within-subjects component are included above. This design required extensive signal averaging of fMRI signals from each subject. Initial (localization) data were acquired from 4 or 5 independent scan sessions, to localize the expected stripes/columns and retinotopy, which was followed by further signal averaging in an additional 4 scan sessions to acquire data for Experiments 3–6. Such extensive data collection was required because of the high spatial resolution (voxels of 1.0 mm^3^) required to reveal (and measure the activity in) the targeted cortical stripes/columns. Specifically, the signal-to-noise ratio is known to decrease as voxel size (a cubic measurement) is reduced, but the signal/noise ratio improves with the square of sample number. Use of a high-field (7T) scanner also improved the signal-to-noise ratio, and the selectivity for small (rather than larger) vessel BOLD signals.

Analysis of all these data required fine-scale coregistration of MR image data across scan sessions, which was greatly facilitated by the FreeSurfer analysis stream. As detailed further below, this approach included the following: (1) automated measurement of (and correction for) head motion, and (2) use of the cortical surface format, which made it possible to define specific regions of interest (ROIs) confined largely to the cortical gray matter sheet (i.e., avoiding voxel sampling in the white matter) and in functionally unrelated banks of adjacent sulci, and from the large pial veins. Reconstruction of each subjects' cortical surface also required further anatomical MR scans.

Subsequent analysis included a grouped-subject component. As detailed below, the effect of each independent variable (measured independently in Experiments 3–6) was measured in each ROI (i.e., each stripe or column type, in each of four areas of interest), and that data were averaged together across all 6–8 subjects, which were matched for gender number (3 or 4 females, respectively).

##### General data analysis.

For all datasets, functional and anatomical MRI data were preprocessed and analyzed using FreeSurfer and FS-FAST (version 5.3; http://surfer.nmr.mgh.harvard.edu/) (Fischl, 2012). For each subject, inflated and flattened cortical surfaces were reconstructed based on the high-resolution anatomical images (Dale et al., 1999; Fischl et al., 1999, 2002).

All functional images were corrected for possible motion artifacts. For each subject, functional data from each run were rigidly aligned (6 df) relative to his/her own structural scan using rigid Boundary-Based Registration (Greve and Fischl, 2009). This approach also facilitated averaging of data collected across multiple scan sessions, for each subject. No spatial smoothing was applied to the imaging data acquired at 7T (i.e., 0 mm HWHM); the 3T retinotopic data were filtered at 2.5 HWHM.

A standard hemodynamic model based on a gamma function was fit to the fMRI signal to estimate the amplitude of the BOLD response. For each subject, the average BOLD response maps were calculated for each condition (Friston et al., 1999). Finally, voxelwise statistical tests were conducted by computing stimulus contrasts based on a univariate general linear model. The resultant significance maps were projected onto each subject's anatomical volumes and reconstructed cortical surfaces.

##### Data analysis (7T).

To reduce the impact of pial surface veins on the evoked BOLD signal, 7T BOLD activity was sampled from the deepest cortical depth. Specifically, for each subject, the gray-white matter interface was first defined based on each subject's high-resolution structural scans using FreeSurfer (Dale et al., 1999; Fischl et al., 1999, 2002). Then the percent fMRI signal change was calculated for each voxel intersecting this surface and projected onto the corresponding vertices of the surface mesh. Given the voxels size used here (1 × 1 × 1 mm), and because BOLD responses are negligible in the white matter, fMRI data from this cortical depth were presumably dominated by BOLD variation in cytoarchitectonic layer 6 and, to a lesser extent, layer 5.

##### Consistency across sessions.

Details of this analysis are similar to those described previously in areas V2 and V3 (Nasr et al., 2016); here the analysis was further applied to areas V3A and V4. The consistency between selectivity maps was compared in a two-split data analysis. In V4, both split sets of data measured the correlation between the fMRI responses evoked by two contrasts of interest (i.e., color vs luminance for the color-selectivity test, and 3D vs 2D for the stereo-selectivity test). In V3A, this analysis was applied only to the stereo-selective test, for reasons described in Results. In both areas, the split sets of data were acquired independently in two different scan sessions, on different days, except in a single instance. In that instance, color-selective data were available for only one scan session from one subject; thus, that specific split analysis was conducted based on alternating runs within the one available session. To minimize concerns that the potential correlation between sessions could be complicated by the nonindependence of activity in adjacent vertices, we pseudo-randomly selected and sampled from only 10% of vertices, measuring the level of correlation between the response strength in corresponding vertices across the two sessions. The resulting correlation values were further compared relative to chance level, defined here as the level of correlation between the two sessions after spatially “shuffling” the vertices.

##### Spatial shuffling.

For each of the four visual cortical areas, we defined an ROI based on the retinotopic representation of stimulus-driven activation. For each of those four ROIs, we changed the registrations between functional and structural scans, and assigned the functional activity to each pseudo-randomly selected vertex within that same ROI, other than the original vertex. This method does not change the overall level of activity within the ROI. Rather, it changes only the spatial distribution of activity. This shuffling was repeated 10,000 times for each subject. The reported *p* values indicate the probability of finding a correlation coefficient that was less than the correlation coefficient of misaligned vertices (i.e., the null hypothesis).

##### Radial versus tangential organization.

To test whether the BOLD maps suggest a radial (e.g., columnar) organization in area V4, we measured the fMRI signal change evoked by the each of the localizing stimuli across cortical layers, for each vertex across the visually activated representation in V4, as described previously in V2 and V3 (Nasr et al., 2016). To increase the contrast-to-noise ratio, this analysis averaged activity across multiple scan sessions. Specifically, we tested for a significant correlation between the color- and disparity-selective activity evoked within deep versus superficial cortical surfaces. As a control, we also measured the level of correlation between activity measured within: (1) each vertex within the deep surface and (2) a randomly selected vertex in the same (deep) surface map that was located 2–3 mm apart from the target vertex along the folded cortical surface. Then the two correlation values were compared for each subject separately, using Fisher's method for comparing correlation coefficients.

##### Functional connectivity.

Details of the functional connectivity analysis are similar to those reported previously (Nasr et al., 2015, 2016). After preprocessing, for each subject, sources of variance of noninterest were removed. These sources of noninterest included all motion parameters measured during the motion correction procedure, the global signal, the mean signal from the portion of ventricles that were included in the acquired EPI slices, and the mean signal from a region within the deep cerebral white matter. Then, the mean BOLD signal time course was extracted for both color- and disparity-selective stripes/columns within V2/V3/V4, to use as seeds in a connectivity analysis. For reasons described below, the mean BOLD signal in V3A was averaged over the activated portion of the area (defined based on the retinotopic mapping for each subject) and then used as the ROI. The correlation coefficient was computed for each of these time course seeds against the preprocessed resting-state time course data, from the rest of the sampled visual cortex. As in most analyses of the stimulus-driven 7T data, for this resting-state analysis the sampling was restricted to voxels intersecting the deep cortical surface.

##### ROI analysis.

All ROIs were defined on the cortical surfaces. Borders of ROIs from V1, V2, V3, V3A, and V4 were defined for each subject based on her/his own retinotopic localizers. In V2, V3, and V4, the location of the thin- and thick-type stripes/columns was based on the results of Experiments 1 and 2. For the ROI analyses of responses in thick-versus-thin-type stripes/columns (but not in the analyses based on retinotopic area), the few sites showing overlapping selectivity for both color and disparity were excluded. Thin- and thick-type stripes (and the retinotopy) were difficult to dissociate around the central visual field, especially in the most retinotopic areas V1 and V2, presumably due to occlusions of the experimental stimuli by the constant fixation target. Accordingly, those very central representations were excluded from further analysis.

##### ANOVA.

All statistical analyses were based on repeated-measures ANOVA. Wherever necessary, results were corrected for violation of the sphericity assumption, using the Greenhouse-Geisser method. Except as noted, data from the left and right hemispheres were averaged together for the ROI analysis, for each subject.

## Results

### Terminology

Specific functional clusters are described here as columns, when that conclusion was supported by evidence in humans based on fMRI (Nasr and Tootell, 2016; Nasr et al., 2016), plus histology (Hockfield et al., 1990; Tootell and Taylor, 1995; Adams et al., 2007), plus much prior evidence in nonhuman primates. Consistent with many studies in nonhuman primates, the more specific term “stripe” is sometimes used for those columns in V2, when described here.

Across a wide range of primate species, the two most active set of stripes in V2 have been identified based on their topographic shape (thin vs thick) in histological tissue stained for cytochrome oxidase, plus functional correlates of these histological markers. Some of these functional preferences (e.g., color vs disparity) have also been used to describe these thin versus thick stripes (respectively), in macaque monkeys (Hubel and Livingstone, 1985, 1987; Tootell and Hamilton, 1989; Ts'o et al., 2001; Xiao et al., 2003) and humans (Nasr et al., 2016). Accordingly, here we used either pair of terms (color vs disparity or thin vs thick) interchangeably in human V2. Here we also extended that terminological interchangeability to areas V3, V3A, and V4, for simplicity and as a more explicit working hypothesis.

### General procedures

fMRI data were acquired from healthy human subjects at high spatial resolution (1 mm isotropic) in a high-field (7T) scanner, in multiple scan sessions in each subject (*n* = 8 subjects in Experiments 1, 2, and 6, and *n* = 6 subjects in Experiments 3–5).

### Experiments 1 and 2: color and disparity selectivity

First, we presented two independent stimulus contrasts that have been shown to selectively activate (and thus localize) stripes/columns in V2 and V3 (see Materials and Methods) (Nasr et al., 2016). To reveal thin-type fMRI activity, Experiment 1 tested for higher responses to gratings varying in color compared with otherwise equivalent gratings that varied in achromatic luminance (see Materials and Methods). Experiment 2 revealed thick-type (disparity-selective) activity, based on RDSs, including either a small binocular horizontal (3D, stereoscopic) disparity, versus an otherwise-identical control RDS presented without binocular disparity (2D).

The resultant maps served as a necessary prerequisite for further experiments, localizing the thin- and thick-type columns in each cortical area of interest, in all 16 hemispheres. Importantly, these stimulus contrasts also furnished primary data, demonstrating the presence of high-resolution color- versus disparity-selective activity in area V4, plus a related effect in V3A (see below).

#### Areas V2 and V3

**Figure 2.**
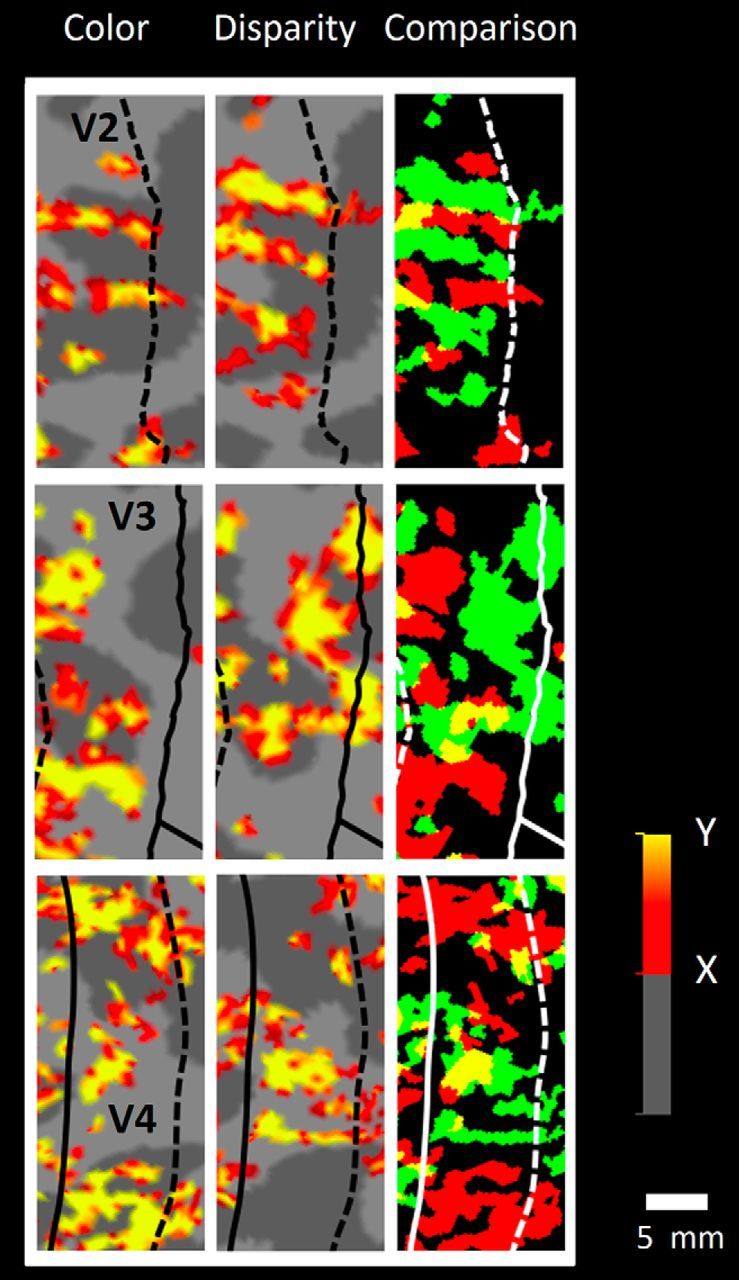
High-resolution fMRI maps of color- versus disparity-selective fMRI activity in visual cortical areas V2, V3, and V4 (top through bottom panels, respectively). All data were acquired at high (1 × 1 × 1 mm) spatial resolution from a 7T scanner. Maps are shown from the lower cortical layers of computationally flattened cortex, based on extensive signal averaging in each subject. Leftmost columns, Color-selective activity (color versus luminance) in each area. Middle, Disparity-selective activity (3D–2D) in the same cortical region. Rightmost panels, Spatial relationship of the color- and disparity-selective maps, at the thresholds used for each of the activity maps. Red represents color-selective sites. Green represents disparity-selective sites. Yellow represents overlap. In all three areas, a tendency for nonoverlap (less yellow) is apparent. Activity is scaled linearly as *p* values between maximum red (*X*; 255, 0, 0) and maximum yellow (*Y*; 255, 255, 0) (bottom right, legend). *p* values higher than *Y* are also encoded as maximum yellow. In V2, these values are *X* = 10^−9^, *Y* = 10^−18^ and *X* = 10^−20^, *Y* = 10^−40^ for the left and middle panels, respectively. In V3, the corresponding values are *X* = 10^−3^, *Y* = 10^−6^ and *X* = 10^−3^, *Y* = 10^−6^. In V4, these values are *X* = 5 × 10^−2^, *Y* = 10^−3^ and *X* = 10^−6^, *Y* = 10^−12^. Area borders are based on independent maps of retintopy for each subject. Solid lines indicate vertical meridian. Dashed lines indicate horizontal meridian. In areas V2 and V3 (top two rows), portions of the graded activity maps (left and middle panels) are also shown in Nasr et al. (2016; their Fig. 14).

Three-color maps (e.g., Fig. 2) at a given threshold supported the model of systematically interdigitated (nonoverlapping) thin-type (color-selective) and thick-type (disparity-selective) stripes in V2, and related columns in V3. Depending on threshold, these maps (Fig. 2, rightmost panels) also showed a few sites of functional overlap (e.g., the yellow-coded regions). It is not yet clear whether such overlapping regions are biologically real or simply a reflection of residual noise in our experimental maps.

**Figure 3.**
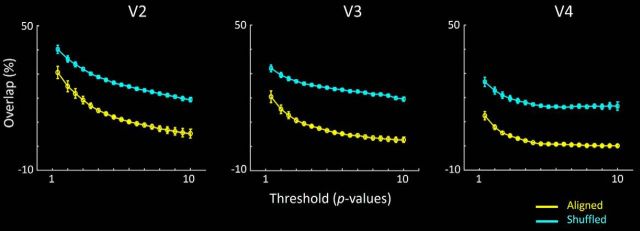
Extent of overlap in the color- and disparity-selective maps, across systematic variations in threshold and topographical alignment, within V2, V3, and V4. Each panel represents group data from all 8 subjects, and both hemispheres. The percentage overlap is calculated as the number of vertices showing color- and disparity-selective activity, relative to the overall number of vertices showing color selectivity or disparity selectivity. Overlap is calculated at multiple threshold levels, across a wide range of *Z* scores (i.e., *p* < 10^−2^ through *p* < 10^−10^). Further, the overlap in color- and disparity-selective activity is calculated when maps were precisely aligned (yellow) or topographically shuffled (cyan). At all thresholds, the percentage overlap was significantly lower when maps were aligned compared with spatially shuffled. Error bars indicate SEM.

These relationships were more systematically measured as a percent overlap (vs nonoverlap) of the two column types, across a wide range of thresholds (see Fig. 3). In the group analysis, the level of overlap between these column types was significantly lower than the chance level, at all thresholds tested, when maps were spatially aligned compared with when maps were spatially shuffled, in areas V2, V3, and V4.

A similar conclusion arose from an analogous analysis across individual hemispheres. Among the 480 possible comparisons (16 hemispheres × 10 thresholds × 3 cortical areas), only three comparisons showed a reversal of the group-averaged result (i.e., more overlap in aligned > shuffled). Moreover, the bias in those three cases was very small, and it was found in only one hemisphere in V2, at the lowest thresholds. Sixteen other comparisons showed no difference in overlap between aligned versus shuffled conditions. The remaining 461 comparisons showed more overlap in the shuffled (compared with aligned) maps.

#### V3A

**Figure 4.**
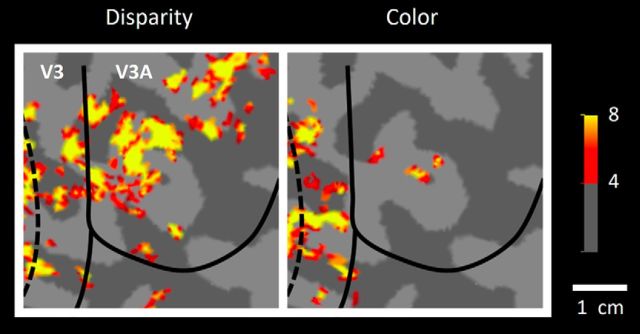
Area V3A is strongly activated by the disparity-selective (but not the color-selective) stimuli. The two maps show color- and disparity-selective activity in V3A, in left and right, respectively, from one hemisphere. A portion of adjacent area V3 is also included. Activity is scaled according to *Z* score. Both panels used a threshold of 4 and a midpoint of 8. Other details are as in Figures 2 and 3.

The functional organization in V3A was strikingly unlike that in V2, V3, and V4. For instance, Figure 4 shows an example of thin- and thick-type (i.e., color- and disparity-selective) activity maps in cortical area V3A. On one hand, consistent with earlier results using conventional fMRI, we found that disparity-selective activity was clustered (Goncalves et al., 2015) and robust (Tsao et al., 2003; Minini et al., 2010) throughout the stimulus-activated region of V3A. Often, we found that such disparity-selective clusters in V3A extended dorsoanteriorly, into higher-level dorsal stream areas within the intraparietal sulcus, consistent with lower-resolution results from conventional fMRI (Tsao et al., 2003; Minini et al., 2010).

**Figure 5.**
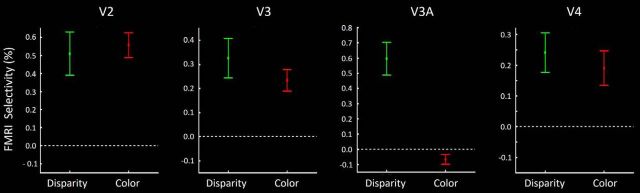
Each panel represents activity sampled throughout the retinotopically activated portion of areas V2, V3, V3A, and V4 (from left to right, respectively), averaged across any columnar variation, from all 16 hemispheres tested. fMRI selectivity for color is the activity difference evoked by color- versus luminance-varying gratings. fMRI selectivity for disparity is the activity difference evoked by 3D versus 2D contrast, in the random dot arrays. Areas V2, V3, and V4 show robust activity to both color- and disparity-selective stimulus comparisons. In contrast, activity in V3A showed robust activity only to disparity-selective stimuli; averaged activity to the color-selective stimuli was near zero.

On the other hand, the color-selective stimuli produced little or no activity in V3A (Fig. 4). This strong dominance of disparity-selective (vs color-selective) activity was found consistently across independent scan sessions, in all 16 hemispheres. Consistent with these observations, a *t* test applied to the level of fMRI activity evoked throughout the retinotopically activated extent of V3A (i.e., averaging across any columnar variation) showed significant selectivity for disparity (*t*_(7)_ = 5.20, *p* < 0.01), but not for color (*t*_(7)_ = −1.90, *p* = 0.10) (Fig. 5). This response pattern was quite unlike the more balanced activity to both color- and disparity-selective localizers, in comparable area-wide analyses in V2, V3, and V4 (Fig. 5). Indeed, color-selective activity was so rare in V3A that we could not reliably test for interdigitated activity between color- and disparity-driven sites, here or below.

This evidence suggested that the functional properties in V3A are similar to those in the thick-type (but not thin-type) columns in V2 and V3. In terms of our hypothesis, this evidence suggests that V3A is strongly influenced by M (but not P) streams. That interpretation was strongly supported in subsequent experiments (see below).

#### V4

As in areas V2 and V3 (but unlike V3A), maps in area V4 showed clustered activity of nonoverlapping color-selective (thin) and disparity-selective (thick) types, plus a few sites of overlap (Fig. 2), depending on threshold. As in V2 and V3, these thin and thick types of V4 clusters were systematically less overlapped compared with chance level (Fig. 3). Unlike the smaller-scale regular alternation of column types observed in V2, color- and disparity-selective clusters in V4 were often grouped into larger-scale sets (e.g., Fig. 2, bottom row).

**Figure 6.**
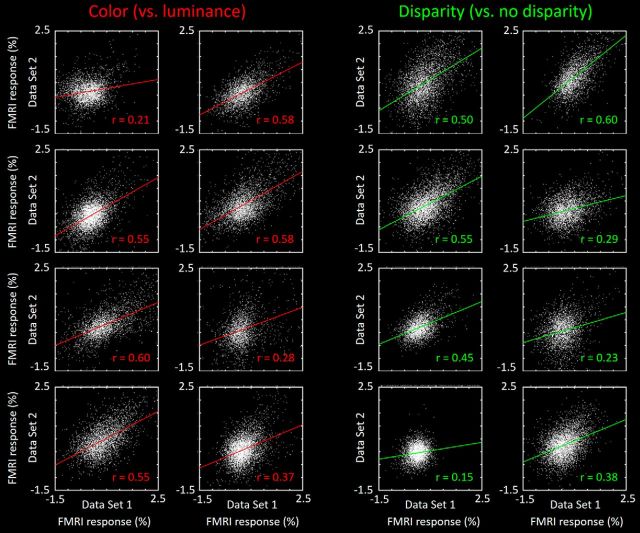
Correlation in BOLD values in maps from V4, shown as split data comparisons in each subject, for responses to both types of localizing stimuli. In each panel, values from data split 1 are shown along the *x* axis; values from the split 2 are shown along the *y* axis. Every white dot represents activity in one vertex of the V4 map, from the deep cortical layers in both hemispheres of each subject, in response to both color- and disparity-selective stimuli (left vs right, respectively). For each subject, data are combined from both hemispheres. Correlation (*r*) values are indicated for each scatterplot.

**Figure 7.**
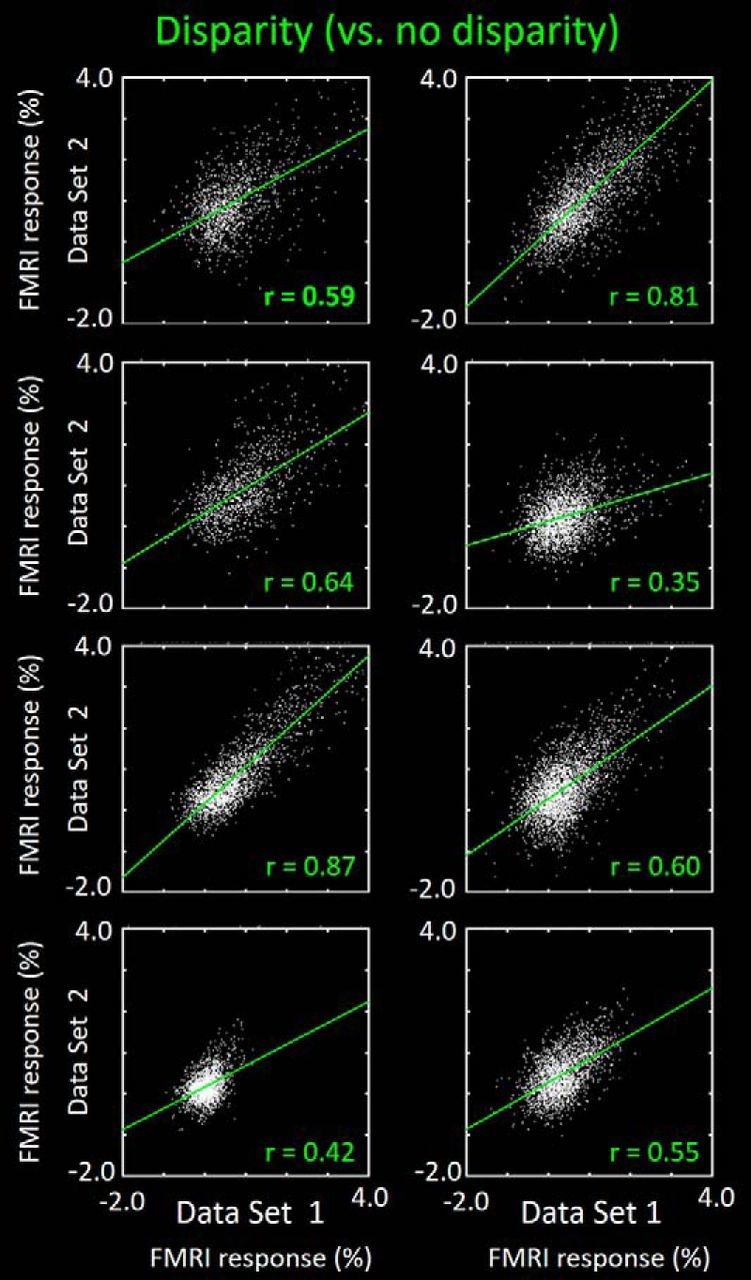
Split data consistency comparison across the cortical maps in area V3A, for the disparity-selective activity. Other details are as in the disparity-selective (right-most, green-coded) activity correlations in Figure 6.

To test the reliability and consistency of the activity maps in V4 (and V3A), we compared the BOLD values in a split data analysis (see Materials and Methods). Results are shown in Figures 6 and 7, for each subject, for V4 and V3A, respectively. In V4, the ROIs were further subdivided into thin and thick (color- and disparity-selective) stripes/columns. In all ROIs, the correlation values were then compared with the correlation values resulting from the same analysis applied to the same sessions, after spatial shuffling the data from one of the maps (see Materials and Methods). To avoid oversampling, we used a permutation method, including only 10% of the vertices (pseudo-randomly chosen) in each comparison, repeating the test 10,000 times. In all subjects, the aligned maps were significantly more correlated to each other compared with the shuffled maps (*p* < 0.01).

**Figure 8.**
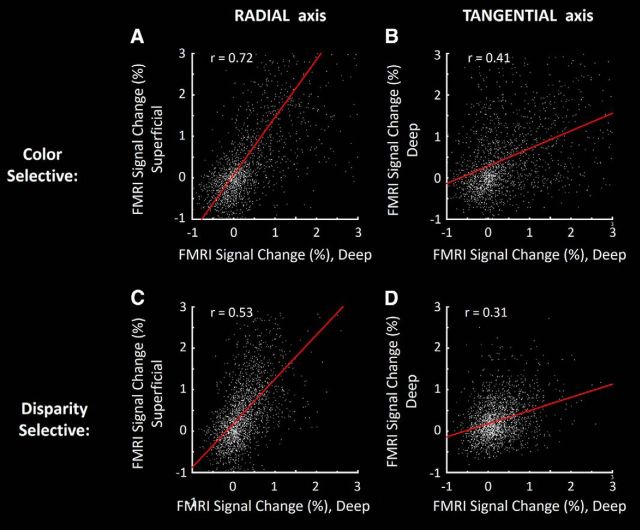
For both color- and disparity-selective activity, a correlation analysis suggests that the BOLD clustering in V4 is strongly columnar in 3D shape, here illustrated in a single subject (both hemispheres). Color-selective activity (***A***, ***B***) is the activity difference evoked by the color versus luminance grating. Disparity selective activity (***C***, ***D***) is the activity difference evoked by 3D–2D contrast, in random dot arrays. ***A***, ***C***, Correlation of activity measured along the radial cortical axis, sampled across deep versus superficial layers in V4 (i.e., along the columnar axis, perpendicular to the cortical surface). ***B***, ***D***, Spatially complementary analysis along the orthogonal (tangential) axis, sampled within the cortical map and within a given (deep) cortical depth. Distances between sampled sites were equivalent, along the radial versus surface-parallel axes. The correlation was significantly higher between BOLD responses sampled within columns compared with across columns (*z* = 24.98), consistent with a strong columnar component in our BOLD maps.

Observations of maps sampled at deep versus superficial layers suggested a columnar arrangement of the functional clusters in V4, as described previously in V2 and V3 (Nasr et al., 2016). To test such observations more systematically in V4, we tested for a BOLD elongation along the radial (columnar) axis. The BOLD ratio of color- and disparity-selective activity was measured in two opposed axes, along an equivalent distance: (1) within a column (measured from superficial vs deep layers) compared with (2) across columns (measured parallel with the cortical surface). The results (Fig. 8) confirmed a prominent elongation of the BOLD activity along the radial axis in V4 (color selectivity: *z* > 10.39; disparity selectivity: *z* > 6.23). That is, the BOLD-based V4 clusters are preferentially columnar in 3D shape.

Given the voxel size used (1 × 1 × 1 mm) and the cortical thickness of V4 in these subjects (2.3140 ± 0.1387 mm), cross-contamination is presumably small or negligible between activity maps measured between deep versus superficial surfaces. Moreover, any such cross-contamination would have presumably reduced the radial bias that we found.

### Experiment 3: luminance contrast

One signature functional distinction between M and P streams is based on contrast sensitivity. Multiple electrophysiological studies in macaque have reported that contrast gain functions differ between M versus P streams, in retina (Kaplan and Shapley, 1986), LGN (Kaplan and Shapley, 1982; Schiller and Colby, 1983; Derrington and Lennie, 1984; Hawken and Parker, 1984), and geniculo-recipient layer 4C of V1 (Blasdel and Fitzpatrick, 1984). In all three sites, M cells showed higher sensitivity to low (e.g., <10%) contrasts, and a shallower (e.g., more saturated) slope at higher contrasts compared with P cells.

Thus, here, our hypothesis predicts analogous contrast gain differences in thick- versus thin-type columns. Accordingly, we tested the contrast gain functions in each column type, in each area. fMRI responses were measured to five different achromatic contrasts, ranging from 1.43% to 99.62% (see Materials and Methods). For each contrast, we averaged together the responses to five spatial frequencies. Changes in the level of stimulus contrast had only a marginal effect on preferred spatial frequency, insignificant at *p* < 0.05 (Table 1).

**Table 1.** Results of application of two-factors repeated-measures ANOVA to activity measured in Experiments 3 and 4 within each ROI

	V2 thin	V2 thick	V3 thin	V3 thick	V3A	V4 thin	V4 thick
Spatial frequency	*F*_(4,20)_ = 36.53	*F*_(4,20)_ = 13.86	*F*_(4,20)_ = 65.19	*F*_(4,20)_ = 40.19	*F*_(4,20)_ = 167.21	*F*_(4,20)_ = 19.19	*F*_(4,20)_ = 126.81
	*p* < 10^−3^	*p* < 0.01	*p* < 10^−4^	*p* < 10^−4^	*p* < 10^−8^	*p* < 0.01	*p* < 10^−8^
Contrast	*F*_(4,20)_ = 13.97	*F*_(4,20)_ = 13.86	*F*_(4,20)_ = 5.21	*F*_(4,20)_ = 8.56	*F*_(4,20)_ = 12.98	*F*_(4,20)_ = 5.05	*F*_(4,20)_ = 6.76
	*p* < 0.01	*p* < 10^−5^	*p* = 0.03	*p* = 0.02	*p* < 0.01	*p* = 0.04	*p* < 0.01
Spatial frequency × contrast	*F*_(16,80)_ = 3.16	*F*_(16,80)_ = 2.80	*F*_(16,80)_ = 1.48	*F*_(16,80)_ = 1.09	*F*_(16,80)_ = 1.58	*F*_(16,80)_ = 3.06	*F*_(16,80)_ = 2.69
	*p* = 0.06	*p* = 0.08	*p* = 0.26	*p* = 0.38	*p* = 0.23	*p* = 0.06	*p* = 0.06

#### V2

**Figure 9.**
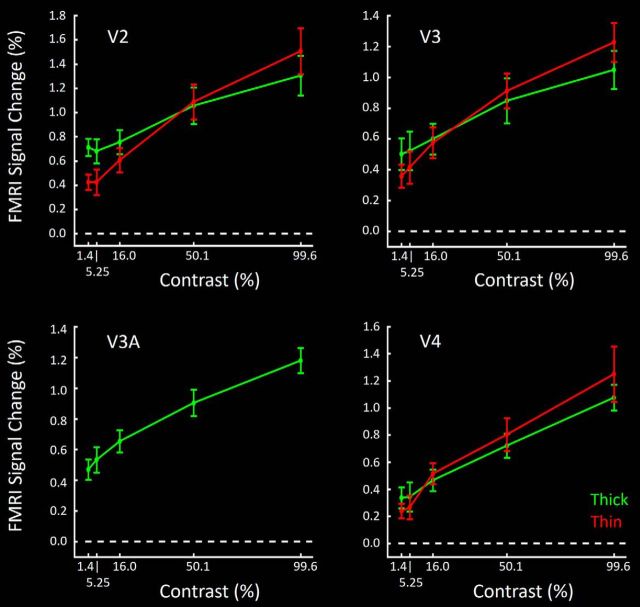
Group-averaged contrast gain functions in thin- versus thick-type columns (red and green, respectively) in area V2 (top left), V3 (top right), and V4 (bottom right). In all three areas, thick-type responses show a higher *y* intercept, and a shallower slope compared with thin-type responses. Because V3A showed few or no color-selective columns, responses in V3A (bottom left) are shown for disparity-selective sites only.

**Figure 10.**
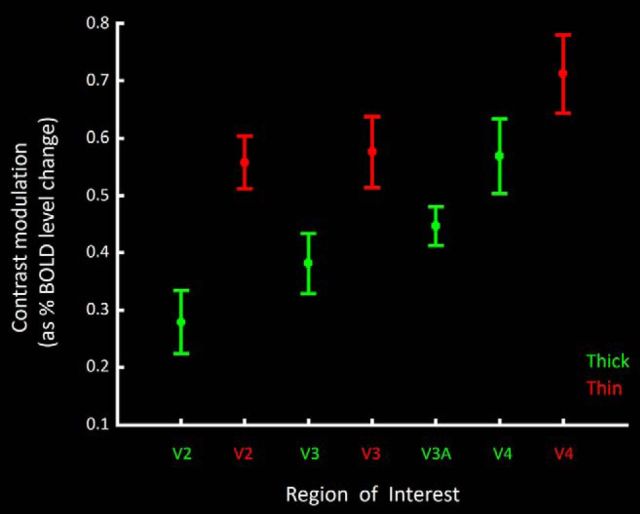
Contrast modulation in thick- and thin-type columns and areas, summarizing the contrast gain data shown in Figure 9. For each ROI, the contrast modulation is calculated as the percentage BOLD response to the stimulus contrasts of the following: (maximum − minimum)/(maximum + minimum). These data confirm a higher-contrast modulation in thin-type compared with thick-type sites, in all three areas that have both types of columns (V2, V3, and V4).

Contrast gain functions in thick- and thin-type stripes (Fig. 9) were generally consistent with predictions from electrophysiological recordings in M and P layers in macaque, respectively. Compared with thin-type stripes, responses in thick-type stripes had lower mean slopes, with relatively higher values at the lowest (1.43%) contrast tested, and relatively lower values at the highest contrasts (99.62%) tested. To more succinctly compare the contrast gain functions, Figure 10 summarized these functions as a single contrast modulation value for each ROI, by calculating the percentage BOLD response to the stimulus contrasts of the following: (maximum − minimum)/(maximum + minimum); maximum and minimum contrasts here were 99.6% and 1.4%, respectively. Consistent with our overall hypothesis, application of a paired *t* test to the measured contrast modulation value showed a significantly higher value in thin- compared with thick-type stripes in V2 (*t*_(5)_ = 5.39, *p* < 0.01).

#### V3

In V3, differences between thin- and thick-type contrast gain functions were similar to those in V2 (Fig. 9). Again, application of a paired *t* test to the measured contrast modulation value (Fig. 10) showed a significantly higher value in V3 thin-type columns compared with thick-type columns (*t*_(5)_ = 6.14, *p* < 0.01).

#### V3A

The contrast gain function in V3A (Figs. 9, 10) was generally as expected from thick-type gain functions at a hierarchical level intermediate between V3 and V4. For instance, the calculated contrast modulation value in V3A was statistically indistinguishable from the value in thick-type columns in V3 (*t*_(5)_ = 1.53, *p* = 0.19) and thick-type columns in V4 (*t*_(5)_ = 1.60, *p* = 0.17) but marginally larger than that in thin-type columns in V3 (*t*_(5)_ = 2.33, *p* = 0.06) and significantly larger than that in thin-type columns in V4 (*t*_(5)_ = 4.45, *p* < 0.01).

#### V4

As in areas V2 and V3, thick-type columns in V4 had a lower calculated contrast modulation value compared with thin-type columns in V4 (*t*_(5)_ = 3.11, *p* = 0.03) (Figs. 9, 10).

### Contrast sensitivity across areas

In addition to the thick-versus thin-type differences in each area, our results suggested a systematic increase in the averaged contrast modulation across areas occupying positions at lower to higher cortical tiers, respectively. Such a hierarchical increase in contrast modulation is consistent with prior data based on conventional human fMRI (Avidan et al., 2002; Yue et al., 2013). This finding is also consistent with conceptual analyses of M- and P-layer single-unit responses in macaque LGN (Purpura et al., 1988). Specifically, in the absence of additional receptive field differences: (1) increased photon catchment increases contrast modulation; (2) photon catchment increases with receptive field size; and (3) receptive field size generally increases with position in the cortical hierarchy.

Consistent with this prediction, we found that independent application of one-way repeated-measures ANOVA (Area: V2 vs V3 vs V4) showed a significant increase in the level of contrast modulation values in thin-type columns from V2 to V3 to V4 (*F*_(2,10)_ = 6.10, *p* = 0.04). Similar application of one-way repeated-measures ANOVA (Area: V2 vs V3 vs V3a vs V4) also showed an increase in the level of contrast modulation values of thick-type ROIs from V2 to V3 to V3A to V4 (*F*_(3,15)_ = 13.90, *p* < 0.01).

### Experiment 4: spatial frequency

Electrophysiologically defined receptive field size and the morphological extent of dendritic arborization are larger in M- compared with P-stream neurons, in macaque retina (Perry and Cowey, 1981; Leventhal, 1982), LGN (Derrington and Lennie, 1984), and V1 (Blasdel and Fitzpatrick, 1984). To the extent that spatial frequency peak tuning scales with receptive field size, our hypothesis predicts that thick-type columns in human visual cortex would show lower peak spatial frequency tuning compared with that found in thin-type columns.

Accordingly, Experiment 4 tested fMRI responses to 1D-varying achromatic sinusoidal gratings, presented at five different spatial frequencies (see Materials and Methods). The range of 1D-varying spatial frequencies tested here (0.1–5.79 c/deg.) was chosen to overlap with, but extend higher than, the range tested in the 2D-varying checkerboard stimuli used in Experiment 2. For each spatial frequency, stimuli were presented at, and averaged across, a logarithmically varied range of five achromatic contrasts (1.43%–99.62%; Experiment 3; see Materials and Methods).

#### V2

**Figure 11.**
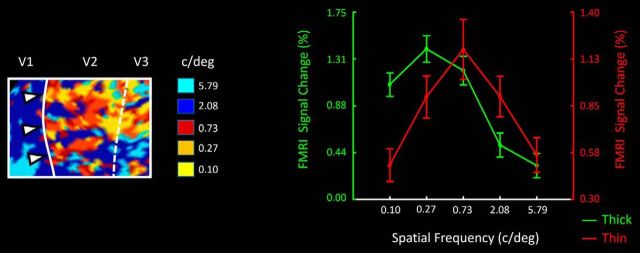
Left, Activity map from V2, in one hemisphere. For each vertex in the map, the preferred spatial frequency (in cycles per degree) is indicated by the color scale shown on the right. The location of thick stripes (based on the disparity-selective localizer) is indicated by right-pointing white triangles. The location of cortical areas V1, V2, and V3 is indicated above the figure, and within the figure as solid and dashed lines, representing the retinotopic location of the vertical and horizontal meridians, respectively. Right, ROI analysis (*n* = 12 hemispheres) showing averaged responses to achromatic, luminance-varying gratings at a range of spatial frequencies. Spatial frequency is indicated on the *x* axis (0.10–5.79 cycles/degree). Responses in thin- and thick-type stripes are indicated in red and green, respectively. BOLD levels for thick- and thin-type stripes are indicated in left and right *y* axes, respectively.

Figure 11 (left) shows a high-resolution map of preferred spatial frequency in V2. Consistent with our hypothesis, this map suggests that thick-type stripes prefer relatively lower spatial frequencies compared with thin-type stripes, within a given local region (i.e., an overlapping retinotopic representation). In the corresponding ROI analysis across all hemispheres, this interpretation was supported by a two-factor repeated-measures ANOVA: Spatial frequency (0.10 vs 0.27 vs 0.73 vs 2.08 vs 5.79 c/deg) and Stripe type (thin vs thick) on the activity measured in thin and thick V2 stripes (Fig. 11, right). Results showed a significant effect of Spatial frequency (*F*_(4,20)_ = 17.12, *p* < 0.01) and, more importantly, a significant interaction between the effects of Spatial frequency and Stripe type (*F*_(4,20)_ = 51.87, *p* < 10^−5^). There was a trend for an effect of Stripe type (*F*_(1,5)_ = 5.33, *p* = 0.07), but it did not reach the *p* < 0.05 level.

#### V3

**Figure 12.**
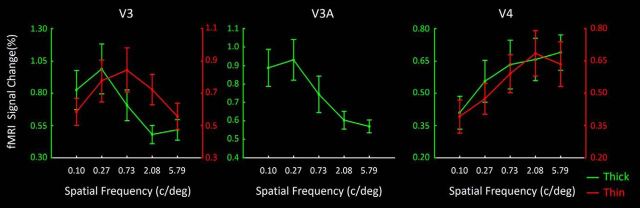
ROI analysis analogous to that in Figure 11, in thin- and thick-type ROIs in area V3 (left), in V3A (middle), and in thin- and thick-type columns in V4 (right). Other details are as in Figure 11 (right).

As in V2, V3 thin-type columns also showed a higher spatial frequency peak compared with thick-type columns (Fig. 12). Again, application of the ANOVA described above showed a significant interaction between the effects of Spatial frequency and Column type (*F*_(4,20)_ = 5.40, *p* = 0.02).

#### V3A

V3A responded most strongly to the lowest spatial frequencies tested (0.1 and 0.27 cycles/degree) (Fig. 12). Application of a one-way repeated measured ANOVA yielded a significant effect of Spatial frequency (*F*_(4,20)_ = 7.15, *p* = 0.03) on activity measured in V3A. The preference for lower preferred spatial frequencies in V3A is again consistent with the interpretation of a dominant M-stream influence, with little or no P-stream influence, as suggested in Experiments 1 and 2.

#### V4

In both thin- and thick-type columns in V4, response functions showed relatively increased activity to correspondingly higher spatial frequencies. However, the functions showed no clear peak (Fig. 12). Accordingly, V4 responses did not show any significant interaction between the effects of Spatial frequency and Column type (*F*_(4,20)_ = 0.55, *p* = 0.60). Instead, we found a significant effect of Spatial frequency (*F*_(4,20)_ = 9.32, *p* < 0.01), without a general effect of Column type (*F*_(1,5)_ = 0.64, *p* = 0.46).

### Experiment 5: chromaticity and 2D spatial frequency

In principle, responses to stimulus color variations are independent of those to spatial variations. However, electrophysiological reports from macaque LGN and V1 suggest that color and spatial sensitivity do interact *in vivo*. For instance, in neurons within P (but not M) layers of LGN, receptive field size is typically larger (and the averaged spatial frequency tuning can be correspondingly lower) when tested with equal luminance color-varying stimuli compared with luminance-varying stimuli, sometimes in the same neuron (De Valois and Pease, 1971; De Valois et al., 1977; Hicks et al., 1983; Derrington et al., 1984; Lennie and Movshon, 2005). Analogous preferences for lower spatial frequencies in response to color-varying (vs luminance-varying) stimuli are reported in human psychophysics (van der Horst and Bouman, 1969; Granger and Heurtley, 1973), and in macaque V1 (Thorell et al., 1984). Thus, if these two M- and P-layer functional properties in LGN remain segregated through mid-level cortex (as hypothesized), one might expect a higher spatial frequency preference in thin-type, but not thick-type, columns, in response to color-varying (relative to luminance-varying) stimuli.

The relationship of color versus spatial sensitivity is also important to clarify to answer another question. Specifically, is the color selectivity found in thin-type columns (e.g., Experiment 1) a general property of human thin-type columns (as in Experiments 1 and 2 and in Nasr et al., 2016), or was that result limited to the specific grating type and spatial frequency that were chosen for Experiment 1?

**Figure 13.**
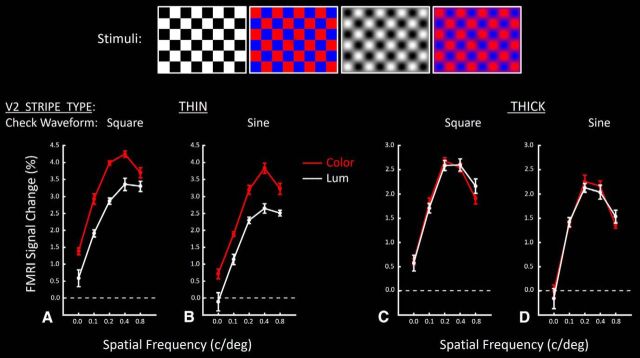
Responses in V2 thin- versus thick-type stripes to contrast-reversing checkerboards. Stimuli were independently varied in three dimensions: (1) chromaticity (color- vs luminance-varying gratings; represented in red vs white functions, respectively), and (2) spatial frequency (along the *x* axis), and (3) waveform (square wave vs sinusoidal checks). Top panels, Four stimulus examples for a given spatial frequency. Square versus sinusoidal checks are shown in the left and right pair of panels, respectively. ***A***, ***B***, Responses in thin-type V2 stripes, relative to a spatially uniform gray baseline stimulus. ***C***, ***D***, Responses in thick-type stripes. ***A***, ***C***, Responses to square wave checks. ***B***, ***D***, Responses to sinusoidal checks.

To test these predictions, we acquired high-resolution fMRI images during presentation of color- and luminance-varying checkerboards, of differing size (locally equivalent to 2D spatial frequencies of 0.00, 0.11, 0.21, 0.42, and 0.84 c/deg), using sinusoidal or square wave checks, independently in different blocks. To maintain robust activation, stimuli were reversed in contrast every 0.5 s. Stimulus examples are shown in Figure 13. The effect of the 2 × 2 experimental manipulations (color vs luminance, i.e., Chromaticity; and sine vs square wave checks, i.e., Waveform) was measured across this range of spatial frequency, in seven ROIs. These ROIs were as follows: (1) thin-type and (2) thick-type stripes in V2, (3) thin-type and (4) thick-type columns in V3, (5) thin-type and (6) thick-type columns in V4, and (7) thick-type clusters in area V3A.

#### V2

In both the thin- and thick-type stripes, the responses to all four checkerboard types (color-varying vs luminance-varying × sine vs square checks) produced systematic, single-peaked tuning functions for spatial frequency (Fig. 13). Application of a three-factor repeated-measures ANOVA (Spatial frequency and Chromaticity and Waveform) showed that activity measured within thin-type stripes responded more strongly to color compared with luminance variations (*F*_(1,5)_ = 99.30, *p* < 10^−3^), across a wide variety of spatial frequencies. Activity in thin-type stripes varied significantly with changes in Spatial frequency (*F*_(4,20)_ = 126.23, *p* < 10^−4^), peaking at frequencies comparable with those used in our localizer gratings (i.e., 0.4 c/deg). In contrast, we found only a marginally significant effect of Waveform (*F*_(1,5)_ = 6.66, *p* = 0.049) on activity evoked in thin-type stripes, without significant interaction between this effect and the effects of the other independent factors (*F* < 0.88, *p* > 0.42). Thus, overall, the color preference found in thin-type stripes in response to 1D-varying sinusoidal grating stimuli of a single spatial frequency (Experiment 1) was here shown to generalize to 2D-varying checkerboard stimuli of both sinusoidal and square waveforms, across a wide range of spatial frequencies.

As hypothesized, we found that V2 thin-type stripes showed a lower spatial frequency peak to color-varying checkerboards compared with luminance-varying versions (Fig. 13). This was evident in a significant interaction between the effect of Spatial frequency and Chromaticity (*F*_(4,20)_ = 6.21, *p* < 0.01).

As expected from the macaque literature, further application of this analysis confirmed that such color biases were absent in thick-type stripes (*F*_(1,5)_ = 0.02, *p* = 0.89). However, instead, we found that responses evoked by color-varying checkerboards were essentially equivalent to responses to the luminance-varying version. That is, the averaged thick-type response did not show a bias for luminance-varying stimuli. A priori, one might instead imagine a luminance bias in thick (or pale) stripes, as a logical counterpart to the color bias found in thin-type stripes, but such a thick-type luminance bias was not found.

Thick-type stripes did not show any significant effect of Waveform (*F*_(1,5)_ = 4.21, *p* = 0.09), and no interaction between the effects of Waveform and Chromaticity (*F*_(1,5)_ = 0.07, *p* = 0.80). In contrast to thin-type stripes, thick-type stripes showed no significant interaction between the effects of Chromaticity and Waveform (*F*_(4,20)_ = 3.09, *p* = 0.09). Insignificant differences were also found with subsequent application of a four-way repeated-measures ANOVA between the two stripe types.

Application of a four-way repeated-measures ANOVA to activity measured within V2 thin- and thick-type stripes showed significant effects of Spatial frequency (*F*_(4,20)_ = 126.45, *p* < 10^−4^), Chromaticity (*F*_(1,5)_ = 36.50, *p* < 0.01), Waveform (*F*_(1,5)_ = 7.20, *p* = 0.04), Stripe type (*F*_(1,5)_ = 83.58, *p* < 10^−3^), Spatial frequency and Chromaticity (*F*_(4,20)_ = 4.72, *p* = 0.03), Spatial frequency and Stripe type (*F*_(4,20)_ = 83.76, *p* < 10^−4^), Chromaticity and Stripe type (*F*_(4,20)_ = 476.77, *p* < 10^−5^), and Spatial frequency and Chromaticity and Stripe type (*F*_(4,20)_ = 4.70, *p* = 0.03). Other effects were insignificant (*p* > 0.10).

Comparisons across thick- and thin-type stripes revealed a strong effect of Spatial frequency (*F*_(4,20)_ = 117.79, *p* < 10^−6^) but no significant interaction between Spatial frequency and Chromaticity (*F*_(4,20)_ = 0.75, *p* = 0.47). This finding suggested a higher peak spatial frequency in thin-type compared with thick-type stripes, in response to both color- and luminance-varying checkerboards. Thus, the difference in V2 spatial frequency peak tuning found in Experiment 4 evidently generalizes broadly across chromaticity and spatial dimensions.

#### V3

**Figure 14.**
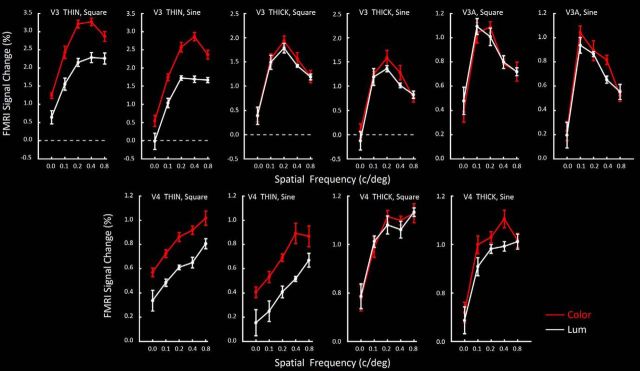
Responses to checkerboard stimuli across a range of spatial frequencies (on the *x* axis) in thin- and thick-type columns, in response to sine and square wave checks, in area V3 and in V3A (top row of panels), and analogous data in V4 (bottom row). Responses to color- and luminance-varying stimuli are indicated as red and white functions, respectively. Other details are as in Figure 13.

Figure 14 shows the analogous response functions in thin- and thick-type columns in V3. As in V2, both thin- and thick-type columns in V3 showed systematic tuning curves for variations in spatial frequency, with little difference in response to square checks compared with sinusoidal checks (Waveform) (Table 2). Moreover, thin-type (but not thick-type) columns in V3 showed a strong bias for color compared with luminance (Chromaticity). Also as in V2, we found a significant effect of Spatial frequency, and an interaction between Spatial frequency and Stripe type between these stripes. Thick-type columns in V3 showed a preference for lower spatial frequencies compared with thin-type columns.

**Table 2.** Results of application of three-factors repeated-measures ANOVA to activity measured in Experiment 5 within each ROI

	V3 thin	V3 thick	V3A	V4 thin	V4 thick
Spatial frequency	*F*_(4,20)_ = 55.59	*F*_(4,20)_ = 64.55	*F*_(4,20)_ = 26.82	*F*_(4,20)_ = 27.91	*F*_(4,20)_ = 45.25
	*p* < 10^−4^	*p* < 10^−5^	*p* < 10^−4^	*p* < 10^−3^	*p* < 10^−6^
Chromaticity	*F*_(1,5)_ = 170.46	*F*_(1,5)_ = 4.55	*F*_(1,5)_ = 0.01	*F*_(1,5)_ = 36.60	*F*_(1,5)_ = 61.18
	*p* < 10^−4^	*p* = 0.09	*p* = 0.97	*p* < 0.01	*p* = 0.06
Waveform	*F*_(1,5)_ = 18.56	*F*_(1,5)_ = 2.00	*F* = 11.54	*F* = 1.01	*F*_(1,5)_ = 0.62
	*p* < 0.01	*p* = 0.21	*p* = 0.02	*p* = 0.36	*p* = 0.47
Chromaticity × spatial frequency	*F*_(4,20)_ = 12.81	*F*_(4,20)_ = 3.21	*F*_(4,20)_ = 0.83	*F*_(4,20)_ = 2.00	*F*_(4,20)_ = 0.88
	*p* < 0.01	*p* = 0.08	*p* = 0.49	*p* = 0.15	*p* = 0.47
Spatial frequency × waveform	*F*_(1,5)_ = 0.16	*F*_(1,5)_ = 0.05	*F*_(1,5)_ = 0.57	*F*_(1,5)_ = 0.39	*F*_(1,5)_ = 0.35
	*p* = 0.79	*p* = 0.99	*p* = 0.54	*p* = 0.62	*p* = 0.66
Chromaticity × waveform	*F*_(1,5)_ = 0.04	*F*_(1,5)_ = 0.12	*F*_(1,5)_ = 1.05	*F*_(1,5)_ = 0.20	*F*_(1,5)_ = 2.59
	*p* = 0.84	*p* = 0.75	*p* = 0.35	*p* = 0.67	*p* = 0.17
Spatial frequency × chromaticity × waveform	*F*_(4,20)_ = 0.52	*F*_(4,20)_ = 0.24	*F*_(4,20)_ = 0.64	*F*_(4,20)_ = 0.23	*F*_(4,20)_ = 0.62
	*p* = 0.62	*p* = 0.74	*p* = 0.54	*p* = 0.81	*p* = 0.53

Application of a four-way repeated-measures ANOVA to activity measured within V3 thin- and thick-type columns showed effects of Spatial frequency (*F*_(4,20)_ = 78.37, *p* < 10^−6^), Chromaticity (*F*_(1,5)_ = 73.64, *p* < 10^−3^), Waveform (*F*_(1,5)_ = 10.53, *p* = 0.02), Stripe type (*F*_(1,5)_ = 19.05, *p* < 0.01), Spatial frequency and Chromaticity (*F*_(4,20)_ = 8.41, *p* < 0.01), Spatial frequency and Stripe type (*F*_(4,20)_ = 18.16, *p* < 0.01), Chromaticity and Stripe type (*F*_(4,20)_ = 400.08, *p* < 10^−5^), and Spatial frequency and Chromaticity and Stripe type (*F*_(4,20)_ = 8.97, *p* < 0.01). Other effects were insignificant (*p* > 0.10).

#### V3A

Figure 14 shows the activity measured in V3A. As in both thin- and thick-type columns in V2 and V3, V3A showed systematically varying responses to differences in spatial frequency, without significant differences between the effects of sine versus square checks (Waveform; Table 2) or other independent factors.

As suggested in Experiments 1–4, the results in Experiment 5 suggested a dominant influence of the M (rather than P) streams in V3A, in two respects. First, V3A responses to color-varying checkerboards were essentially equal to those for luminance-varying checkerboards, as in thick-type columns in V2 and V3. Second, peak spatial frequency tuning in V3A thick-type columns was comparatively low (peaking near 0.1 c/deg), similar to peak responses in thick-type columns in V3 (∼0.2 c/deg).

#### V4

Responses in thin- and thick-type columns in V4 (Fig. 14) were partially consistent with those in thin- and thick-type columns in V2 and V3. Specifically, in thin-type (but not thick-type) columns in V4, color-varying checkerboards produced higher responses compared with luminance-varying checkerboards, consistent with the color preference for sinusoidal gratings of a single spatial frequency in the localizer gratings (Experiment 1). Also, luminance- varying responses were shifted to relatively lower spatial frequencies in thick-type (compared with thin-type) columns. However, direct comparisons were complicated because response amplitudes were generally low, and clear peaks were not evident in most of the V4 thin-type response functions. Instead, the general thin-type pattern was a monotonic increase with spatial frequency throughout the tested range (see Discussion).

### Experiment 6: functional connections

Experiments 1–5 revealed topographically segregated responses to specific, functionally contrasting visual stimuli. Experiment 6 tested for an analogous columnar organization based on functional connectivity, independent of any visual stimuli, from BOLD data acquired during the resting state with eyes closed (see Materials and Methods).

We independently seeded and measured correlations between seeds and targets in the following ROIs: (1) thick-type and (2) thin-type stripes in V2, (3) thick-type and (4) thin-type columns in V3, (5) stimulus-activated regions of area V3A, and (6) thin- and (7) thick-type columns in V4, independently in each hemisphere. Thus, a total of 14 seeds and targets were tested, in all 16 hemispheres.

**Figure 15.**
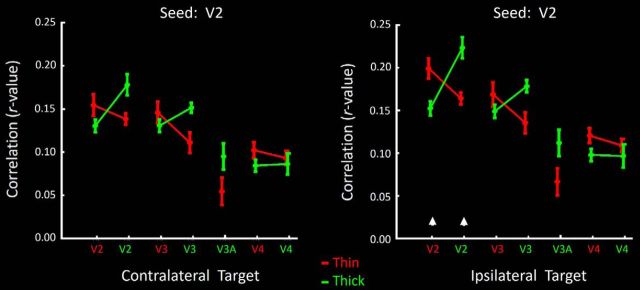
Levels of functional correlation (functional connection) between BOLD fluctuations during eyes-closed conditions using seeds in either thin- or thick-type stripes (red and green functions, respectively) in V2. Levels of correlation with each target location are indicated on the *x* axis; correlation levels are indicated on the *y* axis. Results in contralateral and ipsilateral targets are shown on the left and right, respectively. Results from the seeds and targets in the same area and hemisphere (a control condition) are indicated by white arrowheads above the *x* axis on the right.

**Figure 16.**
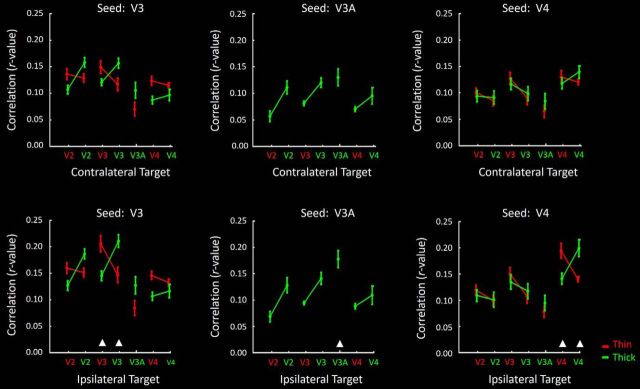
Data from seeds in V3, V3A, and V4; otherwise, same as in Figure 15. Here, results from contralateral and ipsilateral seeds are grouped in the top and bottom rows, respectively. Tables 3 and 4 list the correlation values.

For each of the four areas, we focused on the results from contralateral comparisons, in which all target and seed regions were clearly distinguishable from each other. To clarify, in the subset of ipsilateral comparisons within a common area and column type (Figs. 15, 16, white arrowheads), seeds and targets were (by definition) equivalent, except for the contribution of experimental noise. For that small subset of ipsilateral seeds and targets, the obtained correlation is included only to indicate the level of experimental noise (i.e., how much lower than one is the correlation in this control comparison). Nevertheless, outside of these exceptional seeds/targets, we found that functional connection strength in ipsilateral comparisons was similar to that in contralateral hemispheres.

#### Seeds in V2 thin and thick stripes

Figure 15 (left) shows the level of correlation in activity fluctuations between seeds in V2 thin-type stripes, relative to targets in all ROIs in the contralateral hemisphere. In these contralateral comparisons, activity fluctuations in V2 thin- and thick-type stripes were more strongly correlated between like (compared with unlike) stripe types, as observed previously in fewer subjects (Nasr et al., 2016). Here, application of a two-factor repeated-measures ANOVA [Seed type (thin- vs thick-type stripes) and Target type (thin-type vs thick-type stripes)] showed a significant interaction between these two factors (*F*_(1,7)_ = 9.67, *p* = 0.02).

In further contralateral comparisons, activity fluctuations in V2 thick-type stripes correlated more strongly with V3 thick-type (compared with thin-type) columns. In contrast, V2 thin-type stripes correlated more strongly with V3 thin-type (rather than thick-type) columns. This was evident in a significant interaction between the effects of Seed type and Target type (*F*_(1,7)_ = 5.61, *p* < 0.05). V2 thick-type (compared with thin-type) stripes also correlated more strongly with activity in area V3A (*F*_(1,7)_ = 7.67, *p* = 0.03). However, V2 thick- and thin-type stripe seeds did not correlate selectively with like-type (compared with unlike-type) columns in V4 (*F*_(1,7)_ = 1.10, *p* = 0.33).

In ipsilateral comparisons (Fig. 15, right), similar differences of functional connectivity were found between V2 thin- and thick-type stripes relative to targets in other areas (V3, V3A, and V4), when the V2 stripes were seeded. Specific values are given in Tables 3 and 4.

**Table 3.** Contralateral hemisphere (*p* value for an interaction between the effects of seed type and ROI type)

ROI	Seed			
	V2	V3	V3A	V4
V2	0.02	0.04	0.03	0.33
V3	0.01	0.04	0.04	0.08
V3A	0.02	0.01	—	0.14
V4	0.23	<0.01	0.14	<0.01

**Table 4.** Ipsilateral hemisphere (*p* value for an interaction between the effects of seed type and ROI type)

ROI	Seed			
	V2	V3	V3A	V4
V2	<0.01	0.06	0.03	0.15
V3	0.03	<0.01	0.04	0.06
V3A	0.01	0.03	—	0.21
V4	0.21	0.03	0.26	<0.01

These differences in functional connection could be robust. For instance, the average difference in correlation between like-type (compared to unlike-type) stripes between seeds and targets across hemispheres in V2 (an experimental comparison) was more than half (54%) as large compared with the analogous measurements within the same hemisphere (a control condition; i.e., the maximum possible difference).

Overall, these connectivity results support the evidence from the stimulus-driven experiments (Experiments 1–5) for strongly segregated M-P streams within area V2 (contralateral comparison), and in both hemispheres between V2 and V3, and between thick-type V2 stripes and thick-type clusters in V3A (see below).

#### Seeds in V3 thick- and thin-type columns

Similar results were found after seeding columns in area V3 (Fig. 16; Tables 3, 4). In contralateral comparisons, activity fluctuations between V3 thin- and thick-type columns in a given hemisphere correlated more strongly with fluctuations in like-type (compared with unlike-type) columns in the contralateral hemisphere. Application of a two-factor repeated-measures ANOVA showed a significant interaction between the effects of Seed type and Target type (*F*_(1,7)_ = 6.76, *p* = 0.03).

Activity fluctuations in seeded V3 thin- and thick-type columns also correlated more strongly with like-type (compared to unlike-type) stripes in contralateral V2 (*F*_(1,7)_ = 10.64, *p* = 0.01). Furthermore, fluctuations in V3 thick-type (compared with thin-type) columns correlated more strongly with thick-type columns in area V3A (*F*_(1,7)_ = 6.64, *p* = 0.04). There was a trend for V3 thin- and thick-type columns to be more correlated with like-type, rather than unlike-type, columns in V4, but the interaction between the effects of Seed type and Target type was not significant at the *p* < 0.05 level (*F*_(1,7)_ = 4.16, *p* = 0.08). A significant segregation of functional connections was also evident between V3 thin- and thick-type columns, when these columns were seeded in the ipsilateral hemisphere. Generally, these results again support the stimulus-driven results (Experiments 1–5), suggesting a like-type segregation of functional connections within and between V2 and V3.

#### Seeds in V3A

Given its dearth of thin-type (color-selective) activity (e.g., Figs. 4, 5), we could not test for thin- versus thick-type clustering of functional connections to/from V3A. However, seeding was possible in the activated clusters located within V3A, in contralateral comparisons. Such activity fluctuations in V3A correlated more strongly with those in thick-type (compared with thin-type) columns in V2 (*F*_(1,7)_ = 8.65, *p* = 0.02) and V3 (*F*_(1,7)_ = 10.95, *p* = 0.01), but not V4 (*F*_(1,7)_ = 2.68, *p* = 0.14) (Fig. 16). Similar results were also found when V3A was seeded in the ipsilateral hemisphere (Fig. 16; Tables 3, 4). As in Experiments 1–5, these results suggest that V3A is selectively connected with the M stream.

#### Seeds in V4

As in areas V2 and V3, we found significantly correlated activity fluctuations between seeds in V4 and targets in contralateral V4 (i.e., the interaction between the effects of Seed type and Target type was significant across hemispheres; *F*_(1,7)_ = 12.71, *p* < 0.01). Also, V4 thin- and thick-type columns showed a significantly stronger correlation with like-type (compared with unlike-type) columns in V3 (*F*_(1,7)_ = 14.22, *p* < 0.01).

On the other hand, V4 thin- and thick-type columns showed an equivalent level of correlation with like- and unlike-type stripes/columns in V2 (i.e., the interaction between the effects of Seed type and Target type was not significant; *F*_(1,7)_ = 1.69 *p* = 0.23). Both thin- and thick-type V4 columns were correlated with thick-type columns in area V3A, without significant differences (*F*_(1,7)_ = 2.73, *p* = 0.14).

## Discussion

In macaque monkeys, multiple classic neurobiological techniques have revealed a fundamental and elegant segregation of information processing within magnocellular (M) versus parvocellular (P) streams, in the retina, LGN, and striate cortex, with M-stream influence extending to area MT. From V1, it is not resolved whether: (1) M and P streams project in segregated fashion to different cytochrome oxidase-defined stripes (Livingstone and Hubel, 1987, 1988) or (2) whether V2 stripe differences instead reflect functional differences derived from blob versus interblobs in V1 (Sincich and Horton, 2002, 2005a, b).

**Figure 17.**
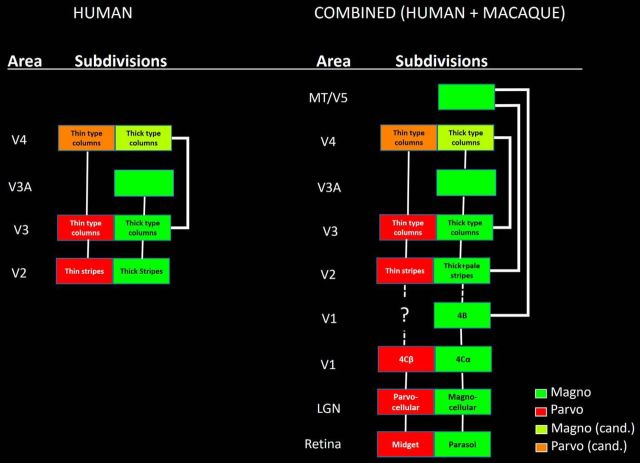
Summary diagrams of the current findings based on fMRI in humans (left), and those results combined with prior results from macaque (right). Other details are as in Figure 1.

Our results suggest that M and P cortical streams exist (and are segregated) in columns in multiple extrastriate areas (Fig. 17, left). Based on high-resolution fMRI combined with multiple types of stimulus selectivity, plus complementary evidence from functional connections in the same subjects, we found a strong segregation of M- versus P-type functions in human V2 and V3. An analogous functional segregation was also evident in area V4, except in tests of spatial frequency. Area V3A was strongly dominated by the M (rather than the P) stream, in all experiments.

Thus, when the current data in humans are combined with the prior data from macaque, the segregation of M- and P-type streams extends from the retina through five or six cortical areas (V1, V2, V3, V3A, MT/V5, and arguably V4 (Fig. 17, right). However as noted above, current evidence does not resolve whether the information in M and P streams extends in an unbroken stream from V1 to V2 (Livingstone and Hubel, 1987, 1988), or instead, whether that information is reorganized into “inter/blob-to-stripe” channels between those two areas (Sincich and Horton, 2002, 2005a, b).

Several studies in macaque also reported a clustered segregation of connections (DeYoe et al., 1994) and function (Tanigawa et al., 2010) in some of the extrastriate areas tested here. Although such clusters were not explicitly tested for M- or P-stream influence, such clusters in monkeys are generally compatible with our current findings in humans.

These combined data suggest an interesting generality: distinct M and P streams (segregated between or within cortical areas) exist throughout the classically retinotopic extrastriate areas; and conversely, M-P segregation weakens in areas in which visual fields are increasingly less retinotopic (e.g., V4). Thus, mechanisms of retinotopy may be linked to mechanisms that maintain M-P segregation. For instance, cortex may tile (replicate) the visual field into duplicated M and P representations for each retinotopic location, as suggested for other columnar variables in V1 (Tootell et al., 1988c) and V2 (Roe and Ts'o 1995; Shipp and Zeki, 2002).

### Area V4

In four experiments (Experiments 1–3, 6), V4 showed evidence for functionally segregated thin- and thick-type columns, consistent with segregated M-P streams. On the other hand, Experiments 4 and 5 found no analogous differences in spatial frequency tuning in V4, and fMRI responses there were low overall. One interpretation is that optimal activation of area V4 requires stimuli that are more complex than either checkerboards or gratings (Gallant et al., 1993; Williford and von der Heydt, 2013). By using such inadequate stimuli here, a hypothetical M and P segregation may not have been evident.

In V4 of macaque, an elegant electrophysiological study (Ferrera et al., 1994) concluded that M and P influences are combined (intermixed) in most neurons. How can that result be reconciled with the evidence for an M and P segregation that we found in human V4? Such differences could arise from species differences and/or from the fundamental differences between data based on fMRI versus electrophysiology. Alternatively, a subtle and/or sparse segregation of M and P inputs may indeed exist in macaque V4. Ferrera et al., (1994) reported two sites in which neurons were significantly dominated by P influences, whereas a different recording site was dominated by M influences.

### Asymmetric sorting

A model of asymmetric sorting was proposed to describe the organization of M-P streams in macaque cortex (Ferrera et al., 1994). That model included the following: (1) a splitting off of the M stream to dominate MT/V5 and additional dorsal stream areas; and (2) a more balanced set of M- plus P-stream information to the ventral stream.

The current evidence extends that model of asymmetric sorting. Such an updated, combined model of M-P streams in humans and macaques (i.e., the two most well-studied catarrhine primates) (Fig. 17, right) suggests that both V3A and MT/V5 are dominated by M-stream inputs, and their ascending output contributes M influences to the dorsal stream. Neurons feeding prominently into the ventral stream show a more balanced combination of M and P influences that are segregated within each area, including thick- versus thin-type columns in V2, V3, and V4.

The current evidence from humans is largely compatible with earlier results from macaque, partly because complementary areas were analyzed in the two sets of data. Prior studies in macaque did not specifically test for M-P segregation in areas V3A or V3. Conversely, here we could not test for M-P segregation in human MT/V5, due to technical constraints.

### Additional functional differences in M-P streams

As initial signature M-P stimulus dimensions here, we chose to test color, binocular disparity, spatial frequency, and contrast. In macaque LGN, M and P neurons may also respond differentially to additional stimulus dimensions. Some of those additional stimulus dimensions may be related to the stimulus dimensions that we tested. For example, it has been noted that the relatively larger receptive fields in M (compared with P) neurons have a larger photon catchment, thus contributing to a higher contrast sensitivity (Kaplan and Shapley, 1982) and/or higher light sensitivity (Purpura et al., 1988) in M (compared with P) neurons. As another example, the subtractive color opponent mechanisms in P (but not M) neurons might also entail a loss of overall light sensitivity (Purpura et al., 1988). In any event, our general hypothesis predicts that thin- and thick-type columns in cortex may show response differences akin to those in LGN, regardless of what those functional properties may be.

Consistent with that hypothesis, Dumoulin et al. (2017) recently reported that the visual stimuli, which were contrast-reversed at two different temporal frequencies, also produced stripe-shaped activity differences in V2 and V3, consistent with histological features of V2 stripes. One caveat is that the temporal frequencies tested in that study (1.5 vs 7.5 Hz) differed from the averaged optimal temporal frequency in parvocellular versus magnocellular LGN layers in macaques (10 vs 20 Hz., respectively) (Derrington and Lennie, 1984).

### Koniocellular cells

This study focused on the functional properties and connections of the principal M and P layers of the LGN. These layers are prominent histologically and physiologically, and historically, much research has focused on these two layers. However, more recently, the role of the very small koniocellular (K) neurons in LGN has also been more explicitly considered (e.g., Hendry and Reid, 2000). Histochemically defined K neurons are very small, located between (and sometimes within) M and P layers of LGN, but the organization and number of K neurons vary markedly across primate species. The functional properties of individual K neurons also differ quite widely, both within and across species. Thus, it is difficult to consider (or manipulate) the specific functional contribution of koniocellular cells as a population (Hendry and Reid, 2000).

Some K cells are influenced by short wavelength (blue) cones, and in macaque, the K cells project directly to the color-responsive blob cells in area V1 (Hendry and Yoshioka, 1994). By that route, K cells could contribute to the color sensitivity found in the V2 thin stripes. However, deoxyglucose labeling showed no obvious sensitivity to color variation (either red, yellow, green, or blue) in K layers of macaque LGN (Tootell et al., 1988b).

### General considerations

Generally, the organization of macaque visual cortex has proven similar to that in humans (Vanduffel et al., 2001; Sereno and Tootell, 2005). This rationalizes the combination of M-P data from humans and primates (e.g., Fig. 17). However, species differences may also exist. For instance, the number of LGN leaflets differs across primate species (Kaas et al., 1978; de Sousa et al., 2013).

At least in macaque, the cortical areas studied here (V2, V3, V3A, and V4) process information partly in an ascending hierarchical sequence, based on laminar analysis of connections between areas (Felleman and Van Essen, 1991; Markov et al., 2014). This hierarchical organization is compatible with models of M-P information flow that emphasize a segregation at lower tiers, then more intermixture in higher tiers. However, many additional connections exist, which may be relevant. For instance, primary visual cortex also projects directly to each of these four areas in parallel (Zeki, 1980; Felleman and Van Essen, 1991; Markov et al., 2014). However, the V1 projections to V3A (Zeki, 1980) and V4 (Yukie and Iwai, 1985; Perkel et al., 1986; Ungerleider et al., 2008) are relatively weak, and retinotopically restricted, so in that sense, the hierarchical model is still supported.
